# Supplementary material for: FeS2/C Nanowires as an Effective Catalyst for Oxygen Evolution Reaction by Electrolytic Water Splitting
Source: Materials (Basel). 2019 Oct 15;12(20):3364. doi: 10.3390/ma12203364 (PMC6829240; doi:10.3390/ma12203364)
Supplement: Supplementary file 1 [file materials-12-03364-s001.pdf]

materials

Supplementary Materials

FeS_2/C Nanowires as an Effective Catalyst for Oxygen Evolution Reaction by Electrolytic Water Splitting

Kefeng Pan [1], Yingying Zhai [2,*], Jiawei Zhang [1] and Kai Yu [1]

[1] School of Metallurgy, Northeastern University, Shenyang 110819, China; xiaopandy@126.com (K.P.); zhang416940558@163.com (J.Z.); yuk@smm.neu.edu.cn (K.Y.)
[2] Computing Center, Northeastern University, Shenyang 110819, China
* Correspondence: zyy@mail.neu.edu.cn; Tel.: +86-1504-008-6983

Received: 10 September 2019; Accepted: 14 October 2019; Published: date

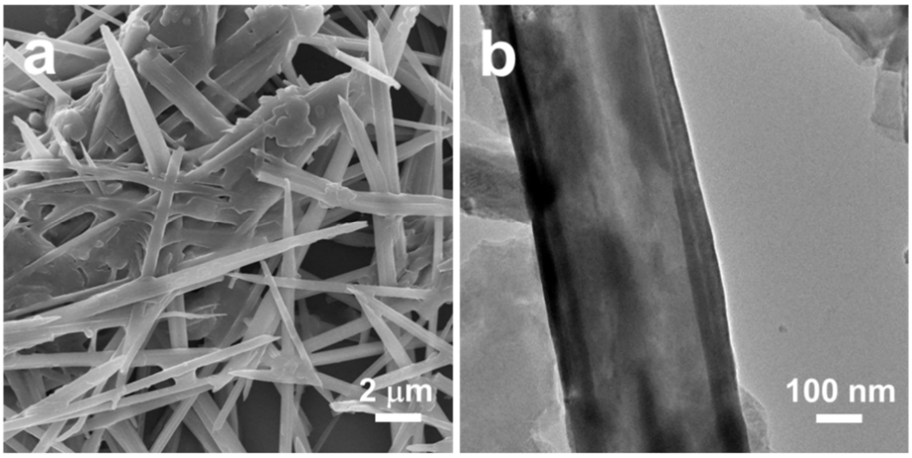

Figure S1. (**a**) SEM and (**b**) TEM images of precursor without the addition of PVP.

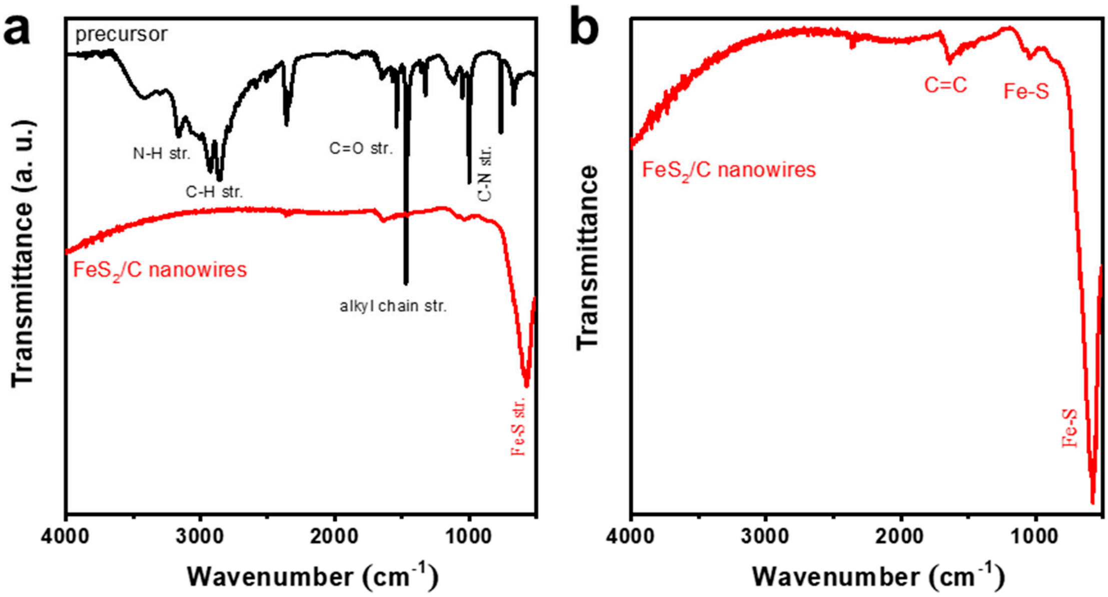

Figure S2. (**a**) FTIR spectrum curves of the precursor and FeS_2/C nanowires, (**b**) enlarged FTIR spectrum curves of FeS_2/C nanowires.

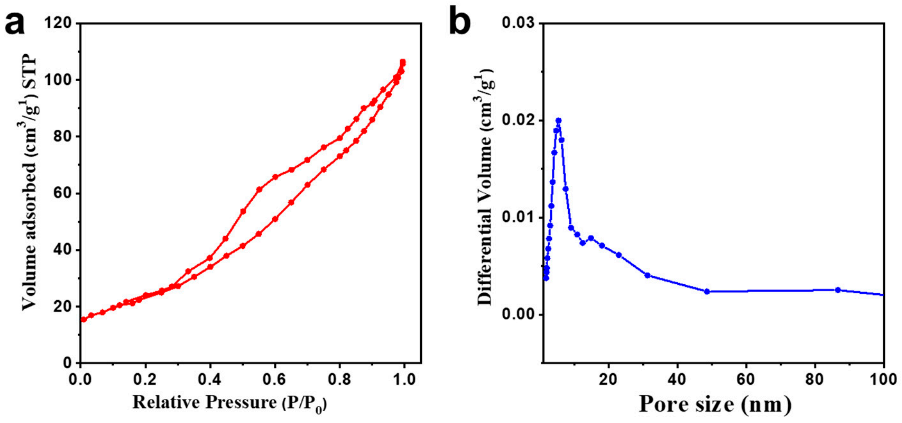

Figure S3. (**a**) N2 adsorption-desorption isotherm and (**b**) pore diameter distribution of FeS2/C nanowires.

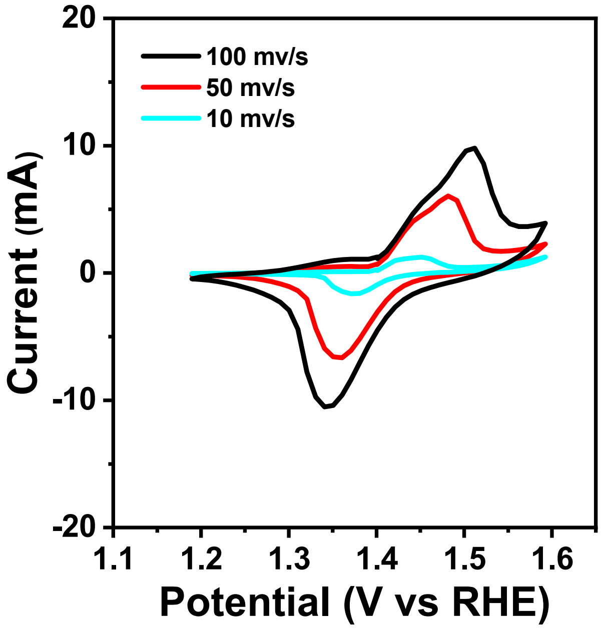

Figure S4. CV curves of FeS2/C nanowires in 1 M KOH at different scan rate of 10, 50 and 100 mV/s.

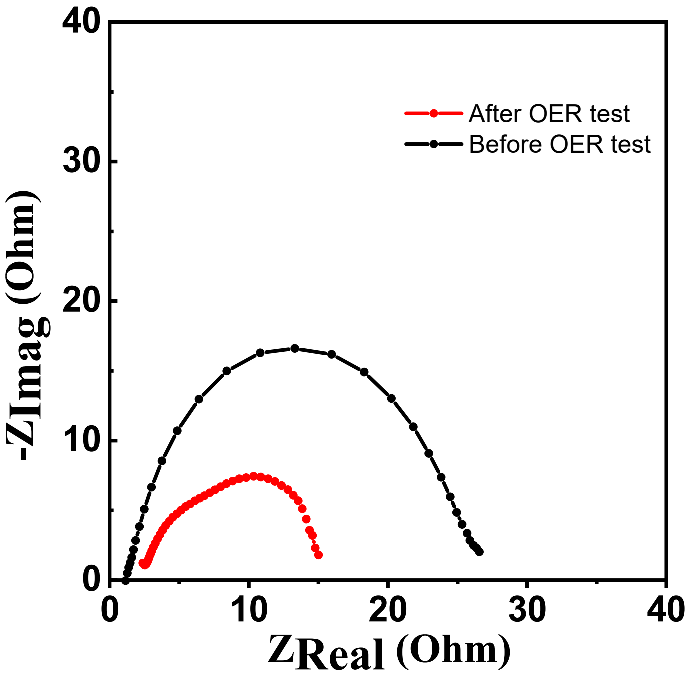

Figure S5. Nyquist plots of the FeS_2/C nanowires before and after OER test in 1.0M KOH.

Table S1. The comparison of catalytic performances for OER in 1 M KOH between the as-prepared FeS_2/C nanowires and other materials reported in the literature.

	Overpotential$_{@10mA/cm2}$ (mV)	Tafel Slope (mV/dec)	Reference
FeS_2/C nanowires	291	65.6	This work
Ni/Mo_xC	328	74	[39]
Fe_3C@NCNT/NPC	339	62	[40]
γ-MoC/Ni@NC	310	62.7	[41]
Fe_3C@NG-800	361	62	[42]
$FeNiS_2$ NSs	310	46	[43]
CP/CTs/Co-S	306	72	[44]

© 2019 by the authors. Submitted for possible open access publication under the terms and conditions of the Creative Commons Attribution (CC BY) license (http://creativecommons.org/licenses/by/4.0/).
